# A Dual-Channel Strain Gauge Force Plate System with Hardware-Triggered Synchronization for Countermovement Jump Analysis

**DOI:** 10.3390/s26134039

**Published:** 2026-06-25

**Authors:** Yue Chen, Guiyang Liu, Yuhao Jia

**Affiliations:** College of Electronic Information and Automation, Tianjin University of Science and Technology, Tianjin 300457, China; 24807924@mail.tust.edu.cn (G.L.); jyh@mail.tust.edu.cn (Y.J.)

**Keywords:** countermovement jump, force plate, strain gauge, hardware synchronization, bilateral asymmetry, test-retest reliability, open architecture, biomechanical measurement

## Abstract

**Highlights:**

**What are the main findings?**

- A dual-channel strain gauge force plate system with hardware-triggered video synchronization achieves stable static consistency (MAPE = 1.01%, R^2^ = 0.9992), low-load linearity (R^2^ > 0.996 at 5.0–30.0 kg), dynamic linearity (R^2^ > 0.997), and cross-modal flight time agreement within ±10 ms of a 240 fps high-speed camera.
- The system demonstrates high test–retest reliability for concentric impulse (ICC = 0.997) and jump height (ICC = 0.987), good reliability for PF (ICC = 0.962) and RFD_100ms_ (ICC = 0.883), and moderate reliability for the bilateral ASI (ICC = 0.748).

**What are the implications of the main findings?**

- The reported minimal detectable change (MDC_95_) for concentric impulse (7.26 N·s) and jump height (1.31 cm) provides preliminary practical thresholds for monitoring general training adaptations and lower-limb neuromuscular function in healthy populations.
- The open-architecture design with fully transparent signal processing and hardware-triggered synchronization enables researchers to customize analysis pipelines (e.g., baseline estimation, filtering, and integration windows) and facilitates multimodal (force-video) data fusion, overcoming the closed-algorithm limitations of commercial systems.

**Abstract:**

Countermovement jump (CMJ) analysis is widely used to assess lower limb neuromuscular function, but commercial force plates often suffer from high cost, closed algorithms, and lack of bilateral independent measurement. This study developed and evaluated a dual channel strain gauge force plate system featuring open architecture and hardware-triggered video synchronization. The system consists of two physically isolated plates, each with four full bridge strain beams, a precision analog front end, and a 2000 Hz acquisition unit. A microcontroller-based hardware trigger synchronizes force data with video capture. Custom host software implements adaptive jump phase recognition and calculates peak force (PF), concentric impulse, jump height, rate of force development (RFD), and asymmetry index (ASI). Validation included static mass measurements in 14 participants, low-load static calibration (5.0–30.0 kg), free-fall impulse validation (7.00 to 31.32 N·s), 240 fps high-speed video cross validation of flight time, ecological-validity comparison with published AMTI-based force-plate data, and 48 h test–retest reliability assessment. Static mass measurement showed a mean absolute percentage error (MAPE) of 1.01% and a coefficient of determination ($R^{2}$) of 0.9992, while low-load testing confirmed excellent linearity ($R^{2}>0.996$) and minimal absolute error (mean absolute error = 0.34 kg) at lighter weights. Dynamic impulse validation yielded $R^{2}>0.997$ and MAPE < 3%. Flight time agreement with high-speed video was within ±10 ms. Test–retest reliability was excellent for concentric impulse (intraclass correlation coefficient (ICC) = 0.997) and jump height (ICC = 0.987), and good for PF (ICC = 0.962) and rate of force development at 100 ms (RFD_100ms_) (ICC = 0.883). The physically isolated dual-plate architecture effectively captured bilateral force differences, although the ASI demonstrated moderate reliability (ICC = 0.748), likely reflecting the inherent biological variability in bilateral coordination. The ecological-validity comparison further indicated that the macroscopic kinetic outputs of the proposed system fell within the expected physiological and biomechanical ranges reported for adult CMJ testing. Overall, these findings support the study hypothesis that the proposed dual-channel force plate system provides a valid, reliable, and cost-effective solution for synchronized bilateral CMJ kinetic assessment in sports performance monitoring and biomechanical research, while offering improved accessibility through an open-source and transparent analysis framework with a hardware cost below 500 USD.

## 1. Introduction

Accurate measurement of ground reaction force (GRF) and its associated kinetic variables is fundamental to quantitative biomechanics research [1,2,3]. In countermovement jump (CMJ) testing, force plates provide key metrics—such as jump height, concentric impulse, peak force (PF), rate of force development (RFD), and asymmetry index (ASI)—which are extensively used to evaluate lower limb explosive power, neuromuscular function, and training adaptability [4,5,6,7]. Therefore, developing scalable force measurement systems with good measurement consistency, dynamic response capability, and practical accessibility remains highly valuable [8].

A force plate’s performance is jointly determined by its mechanical design, analog front-end (AFE), and digital signal processing [9,10]. While previous studies have optimized individual modules—such as using finite element analysis (FEA) to reduce structural crosstalk [11,12,13] or improving bridge circuits to enhance the signal-to-noise ratio [14,15]—research on comprehensive hardware-software co-design remains limited [16]. For sport biomechanics, system-level integration of the underlying architecture and top-level algorithms is critical to achieving stable high-frequency dynamic acquisition and cross-modal synchronization.

Beyond traditional resultant GRF analysis, extracting independent bilateral force characteristics is essential for evaluating neuromuscular control, identifying bilateral asymmetries, and tracking training adaptations [17,18,19]. Conventional single-plate platforms cannot easily separate left and right lower limb force production, limiting their utility in rigorous ASI assessment [20,21]. Consequently, there is a clear demand for force platforms with independent bilateral measurement capabilities.

Furthermore, cross-modal time synchronization of kinetic data and kinematic video is increasingly vital for comprehensive biomechanical analysis [22,23]. Existing force-vision synchronization solutions typically rely on either prohibitively expensive proprietary motion capture systems or tedious post hoc manual frame alignment [24,25]. Manual alignment is not only labor-intensive but also prone to subjective errors [26], which compromises the precise identification of key transient events in fast actions like the CMJ [27,28]. Thus, achieving stable hardware-level time synchronization remains a critical challenge.

Commercial force platforms such as Kistler and AMTI are widely used in research and practical applications [29,30]. Although these systems offer excellent measurement performance and engineering maturity, their high cost, dependence on proprietary hardware and software ecosystems, and limited access to low level interfaces and algorithms may restrict flexibility for customized experimental tasks [31]. Therefore, developing a customized force platform with an open architecture and transparent algorithms is of practical significance for experiments requiring flexible triggering, adaptable data processing workflows, and specialized analysis functions.

To address the limitations of existing commercial force measurement systems in cross-modal synchronization, algorithm transparency, and cost-effective bilateral independent measurement, this study proposes a dual-channel strain gauge force plate system with open architecture and hardware-level video trigger synchronization. The system is intended to enable synchronized kinetic and visual data acquisition while supporting flexible hardware integration and data processing. Its physically isolated dual-plate design further allows independent bilateral force measurement, providing a structural basis for asymmetry analysis and lower-limb functional assessment. For the system’s quantitative validation, the CMJ was exclusively selected because its complex stretch-shortening cycle rigorously tests continuous force tracking and adaptive phase segmentation. Although the system hardware is fully capable of capturing other jump variations, such as squat jumps (SJs) and drop jumps (DJs), these were intentionally omitted to keep the validation strictly focused on the CMJ algorithm and to avoid diluting the methodological objective of this study. Overall, this work aims to provide a cost-effective and experimentally accessible multimodal measurement platform for sports biomechanics and related movement-science research applications.

## 2. Materials and Methods

### 2.1. System Overview

**Figure 1:**
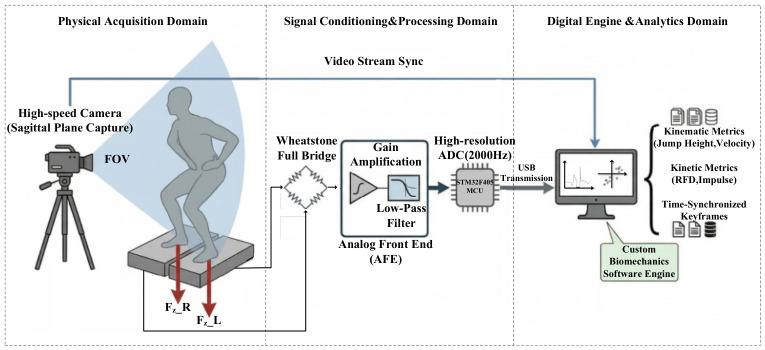
Overall architecture of the synchronized biomechanical analysis system.

This study constructed a dual-channel force measurement system for multimodal CMJ analysis, with its overall architecture shown in Figure 1. The framework comprises the following three functional domains: (a) physical acquisition of vertical ground reaction force (vGRF) and sagittal plane kinematics during CMJ testing; (b) signal conditioning based on AFE and digitization by the microcontroller unit (MCU); and (c) custom software engine for real-time time synchronization and kinematic variable extraction. The system mainly consisted of left and right independent dual-plate mechanical acquisition units, a hardware-triggered video acquisition module, and a host computer data processing module. During testing, the dual plates independently recorded bilateral vGRFs, while a lateral camera captured sagittal-plane motion for synchronized analysis. The force signals were conditioned, sampled at 2000 Hz by the main control unit, and synchronized with the video data through hardware triggering combined with timestamp correction. Subsequently, the host computer performed phase demarcation of the jumping action based on the characteristics of the force–time curve and automatically calculated metrics such as PF, concentric impulse, jump height, RFD, and ASI.

### 2.2. Force Plate Design and Signal Chain

#### 2.2.1. Mechanical Structure

**Figure 2:**
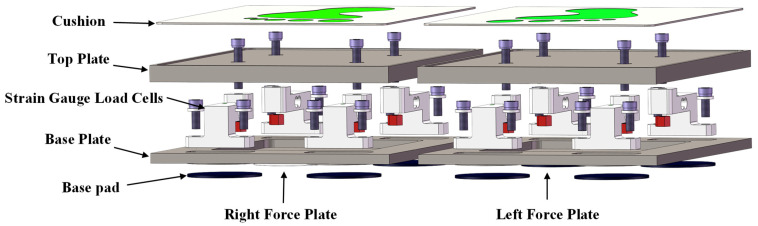
Schematic diagram of the mechanical structure of the force plate.

The proposed system consists of two mutually independent, single-axis force measurement base plates. Each plate adopts a symmetrical “upper load-bearing platform–strain beam–lower support platform” sandwich structure, with its overall configuration shown in Figure 2. Both platforms are machined from structural steel to ensure high structural stiffness and minimize additional deformation during load transfer.

**Figure 3:**
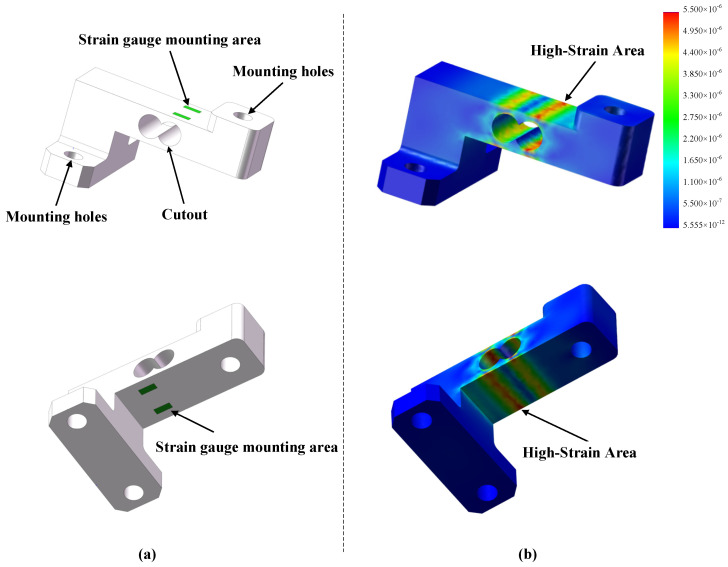
Structural design and mechanical simulation validation of the cantilever force sensor: (**a**) CAD model of the sensor assembly; (**b**) local FEA strain distribution map of a single beam under a total system load of 40 N.

At the core of the force-to-electricity conversion are four custom cantilever-type aluminum alloy strain beams located at the corners (Figure 3a). To optimize the balance between load capacity and measurement sensitivity, a local thinning cutout was introduced near the neutral axis of each beam, leveraging stress concentration to amplify local strain [32], as verified by FEA under a representative 40 N vertical load (Figure 3b).

**Figure 4:**
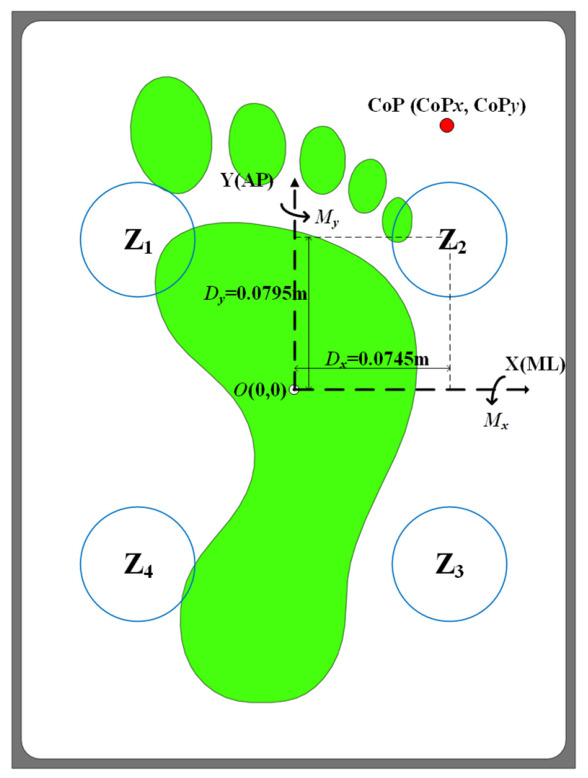
Spatial mapping relationship of the orthogonal coordinate system for a single plate.

To establish the mechanical model of a single force plate, a local coordinate system was defined at the geometric center of the platform following standard biomechanical conventions [33], shown in Figure 4. Based on the vertical forces measured by the four corner sensors, the system calculates the total vertical force and orthogonal moments to estimate the Center of Pressure (CoP). Approximate CoP trajectories were used only for stance-centering guidance and data quality monitoring and were excluded from quantitative biomechanical analyses.

#### 2.2.2. Signal Conditioning Chain

To minimize electrical noise and ensure stable component operation, separate analog and digital power domains were adopted. Each strain beam utilized a full Wheatstone bridge topology to maximize output sensitivity and provide inherent temperature compensation [34]. The microvolt-level differential outputs from the bridges were first filtered and then amplified using precision INA828 instrumentation amplifiers (Texas Instruments, Dallas, TX, USA) with a set gain of 501. To prevent aliasing during subsequent analog-to-digital conversion [35], the amplified signals were passed through a second-order Sallen–Key low-pass filter network with a cutoff frequency of 1000 Hz. Following analog conditioning, the signals were digitized by a 16-bit analog-to-digital converter (ADS8331, Texas Instruments, Dallas, TX, USA) via a high-speed SPI interface.

### 2.3. Data Acquisition and Cross-Modal Synchronization

**Figure 5:**
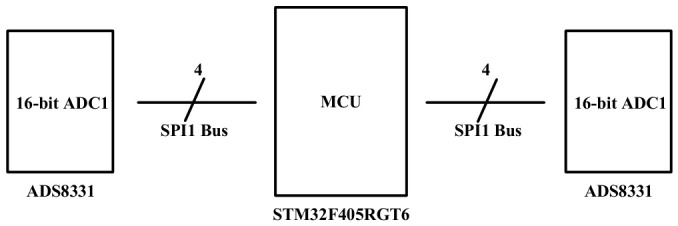
High-speed acquisition module.

To ensure precise temporal alignment between kinetic signals and visual kinematics, the system implemented a hardware-in-the-loop synchronization mechanism. The MCU (STM32F405RGT6, STMicroelectronics, Geneva, Switzerland) continuously sampled eight channels of force data at 2000 Hz (Figure 5). Simultaneously, an internal hardware timer generated a trigger signal to control the external camera’s exposure at 60 fps. A hardware frame index was simultaneously embedded into the force data stream to establish a unified temporal reference between force and video acquisition.

**Figure 6:**
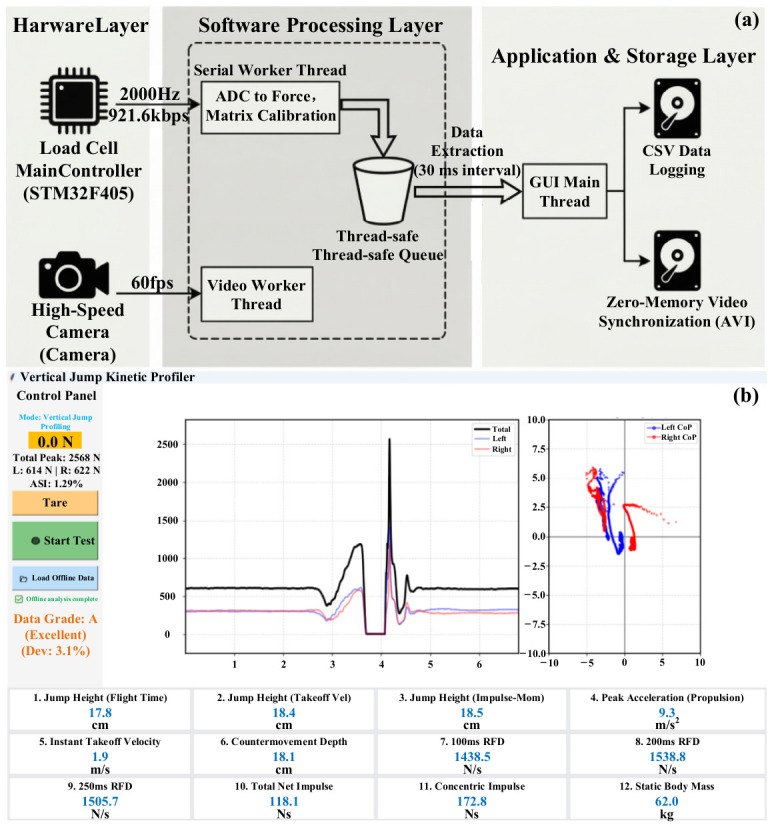
Overview of the system software architecture and GUI: (**a**) software processing layer and data pipeline; (**b**) screenshot of the main GUI.

A custom host program based on Python (version 3.9, Python Software Foundation, Wilmington, DE, USA) managed high-speed serial communication, synchronized video acquisition, real-time visualization, and offline kinetic analysis (Figure 6). Video frames were aligned to the 2000 Hz force timeline using nearest-neighbor timestamp matching with fixed exposure-delay compensation (1.456 ms). Furthermore, the approximate CoP coordinates were rendered as real-time scatter points on the GUI, serving strictly as a qualitative auxiliary tool for pre-test stance-centering control to reduce extreme eccentric loading crosstalk.

### 2.4. Data Processing and Kinetic Variable Extraction

#### 2.4.1. Signal Calibration and Conditioning

**Figure 7:**
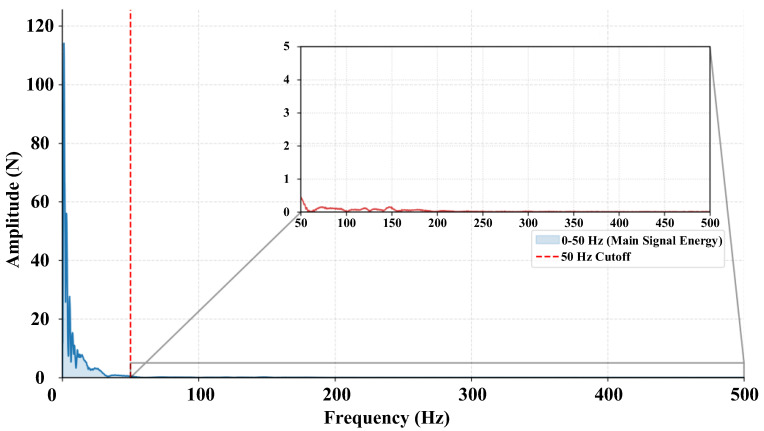
FFT amplitude–frequency spectrum of a raw CMJ force signal.

To eliminate systematic errors from eccentric dynamic loading, a multiple linear regression model was applied to extract a decoupled system sensitivity matrix based on the joint four-channel voltage responses. For digital conditioning, raw force signals were processed using a 50 Hz notch filter followed by a fourth-order zero-phase Butterworth low-pass filter (50 Hz cutoff) [36], based on fast Fourier transform (FFT) analysis of CMJ signal frequency content (Figure 7).

#### 2.4.2. Automated Phase Demarcation and Parameter Calculation

**Figure 8:**
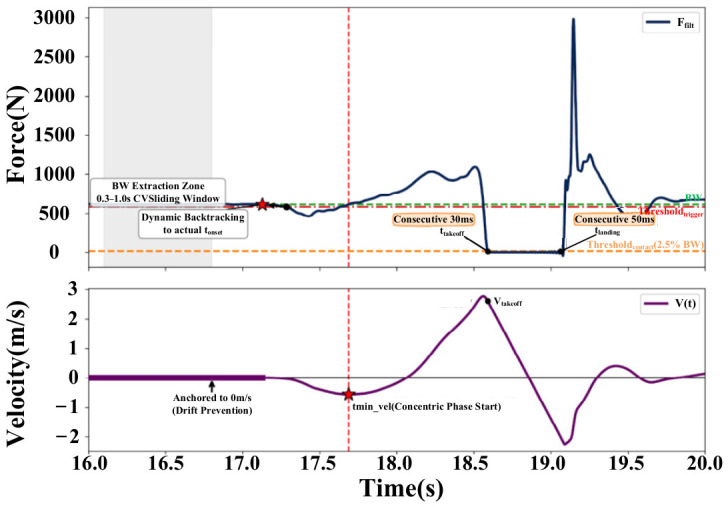
Identification of jump characteristic events and kinetic calculation curves.

An automated, data-driven workflow was constructed to identify jump characteristic events (Figure 8). Prior to jump initiation, resting body weight (BW) and baseline noise standard deviation (${\mathrm{SD}}_{\mathrm{BW}}$) were estimated using an adaptive sliding window algorithm. The onset of the downward eccentric phase ($t_{\mathrm{onset}}$) was triggered when the force curve first dropped below $\mathrm{BW}\text{-}\max\;(5\;\times \;{\mathrm{SD}}_{\mathrm{BW}},\;15.0\;N)$, followed by a backward search to locate the exact force production start point. Takeoff ($t_{\mathrm{takeoff}}$) and landing ($t_{\mathrm{landing}}$) instants were detected using max (BW × 2.5%, 15.0 N) contact threshold, supported by a consecutive-frame validation window to prevent false triggers from mechanical vibration. The center of mass velocity was calculated by integrating the net vertical force, forcing the velocity before $t_{\mathrm{onset}}$ to 0 m/s to prevent integration drift. After the precise temporal phase demarcation of the jump is completed, the system automatically extracts 12 biomechanical parameters from the filtered force-time curve and velocity curve. The definitions for these core variables are explicitly outlined as follows: (1) Concentric impulse was calculated as the area under the net vertical force-time curve during the upward propulsion phase; (2) Jump height (*h*_imp_) was derived using the impulse-momentum theorem, with the integration strictly initiated from the onset of the crouching motion (unweighting phase) to takeoff; (3) PF was defined as the maximum vertical ground reaction force recorded during the entire jump phase; (4) RFD_100ms_ represented the rate of force development over the first 100 ms of the concentric phase; and (5) ASI was calculated to quantify the bilateral percentage difference between the left and right force plates.

#### 2.4.3. Postural Cross-Validation of Jump Height

To improve the reliability of jump height calculation, the system simultaneously adopts three biomechanical methods for independent calculation [27]:

- Flight time method:
  (1) $$h_{\mathrm{flight}}=\frac{1}{8}g\cdot {\left(t_{\mathrm{landing}}-t_{\mathrm{takeoff}}\right)}^{2}.$$
- Takeoff velocity method:
  (2) $$h_{\mathrm{vel}}=\frac{{v_{\mathrm{takeoff}}}^{2}}{2g}.$$
- Impulse–momentum method:
  (3) $$h_{\mathrm{imp}}=\frac{1}{2g}{\left(\frac{1}{m}\int_{t_{\mathrm{onset}}}^{t_{\mathrm{takeoff}}}(F_{\mathrm{filt}}(t)-\mathrm{BW})dt\right)}^{2}.$$

Because $h_{\mathrm{flight}}$ is highly sensitive to aerial posture variations, whereas $h_{\mathrm{vel}}$ primarily reflects the mechanical output during takeoff, the discrepancy between $h_{\mathrm{vel}}$ and $h_{\mathrm{flight}}$ was used as an indicator of postural compensation or abnormal jump execution [27].

**Figure 9:**
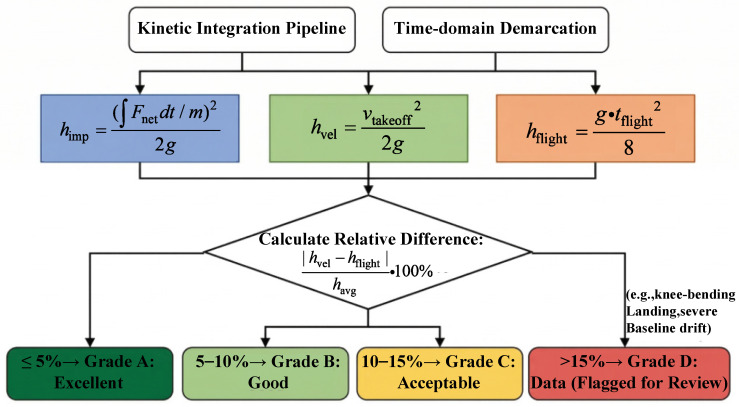
Cross-validation of jump height using multiple physical laws and automatic data quality rating process.

Based on this principle, the following automated data quality grading system was established: Grade A (≤5%), Grade B (5–10%), Grade C (10–15%), and Grade D (>15%) (Figure 9). The ≤5% criterion for Grade A was adopted from the methodological recommendation proposed by Kibele [37], whereas the wider Grade B–D intervals were empirically defined based on pilot observations and routine field-testing experience. Trials classified as Grade D (>15%) were considered to indicate substantial postural compensation or integration drift and were automatically flagged as invalid for retesting.

For all valid trials, *h*_imp_, derived from the impulse–momentum method, was used as the final kinetic outcome to minimize the influence of aerial posture variations.

## 3. Simulations

### 3.1. Finite Element Simulation of the Structure

**Figure 10:**
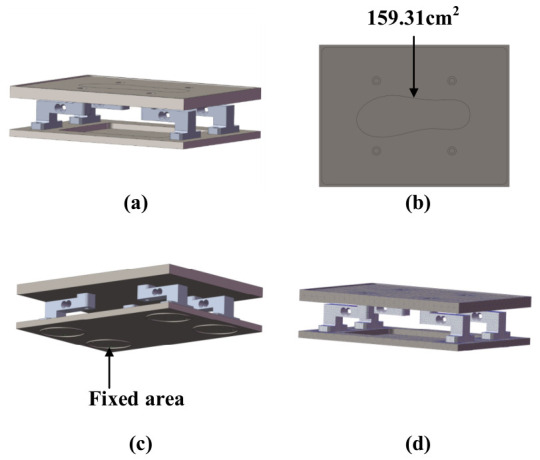
Finite element model setup of the force plate: (**a**) three-dimensional solid model; (**b**) equivalent active footprint area for dynamic loading; (**c**) fixed support boundary conditions at the bottom; (**d**) mesh strategy with local refinement for the strain gauge attachment region.

A three-dimensional solid finite element model was constructed in this study (see Figure 10a) to systematically evaluate the static and transient dynamic characteristics of the force plate. In the model, the load-bearing platform and the strain beams were made of structural steel and aluminum alloy, respectively (mechanical properties are shown in Table 1). A bonded contact constraint was set between all components to accurately simulate the actual bolted fastening state.

Unlike the traditional simplified treatment of a uniformly distributed load over the entire plate, this study introduces realistic biomechanical boundary conditions as follows: referencing the dynamic plantar characteristics of professional basketball players, a non-uniformly distributed load was applied with an equivalent unilateral lower limb contact area of 159.31 cm^2^ [38] (Figure 10b), to accurately reproduce the local pressure surge phenomenon under realistic gait conditions. Regarding boundary and load condition settings, a fully fixed constraint was applied to the four circular support surfaces at the bottom of the model (Figure 10c). For the mesh discretization strategy, the overall structure was meshed using high-order tetrahedral solid elements. To maximize the strain calculation accuracy in the core sensing region while controlling the overall computational cost, local mesh refinement was specifically performed on the thinned region of the cantilever beam (i.e., the preset strain gauge attachment location) (Figure 10d).

#### 3.1.1. Static Analysis

A single support beam was selected as the analysis object. Static forces of 0 N, 400 N, 800 N, 1200 N, 1600 N, 2000 N, 3000 N, 4000 N, and 5000 N were sequentially applied to the equivalent loading area at the center of the upper surface of the force plate. The average stress values in the four strain gauge attachment regions on the strain beam were recorded to verify the linear response and stress distribution characteristics of the proposed structure (see Table 2).

**Figure 11:**
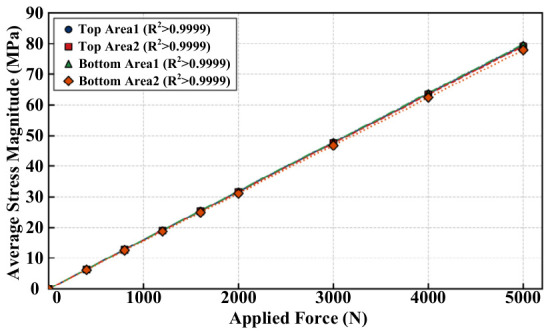
FEA-based stress-force linear relationship.

As shown in Figure 11, the applied force and the simulated stress exhibit a high degree of linearity, with coefficient of determination $(R^{2})\geq 0.9999$ for the four sensing regions. Further calculation of nonlinear error confirms that the maximum relative deviation in each region is less than 0.01% F.S. Moreover, at the maximum applied load of 5000 N, the average stress in the sensing regions is only about 10% of the material’s yield strength. This effectively demonstrates that within the 5000 N loading range of a single force plate, the strain beam strictly follows Hooke’s law, remains entirely in the pure elastic working region, and does not undergo any nonlinear plastic yield, thereby providing a reliable physical premise for the system’s wide-range extrapolation calibration.

#### 3.1.2. Dynamic Characteristic Analysis

The dynamic characteristics of the force plate directly determine the acquisition accuracy of human jump kinetic data and the ability to resist structural resonance. The finite element modal analysis results show that the first natural frequency of the system is approximately 206 Hz (as shown in Table 3), which can effectively avoid the typical excitation frequency band of conventional human movements.

#### 3.1.3. Transient Dynamic Simulation

**Figure 12:**
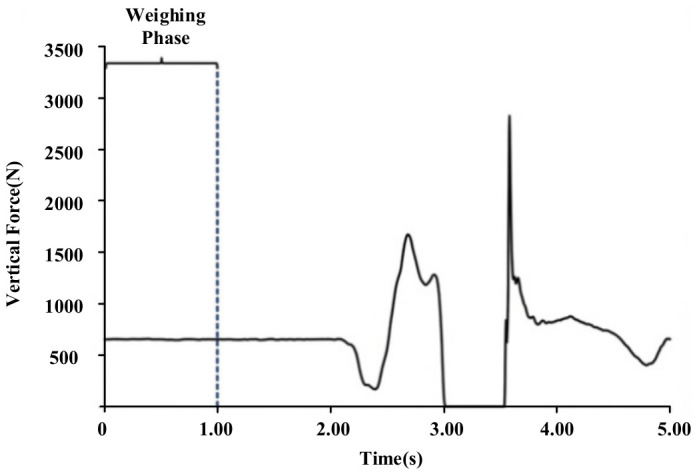
Vertical jump force curve [39].

Based on dynamic characteristics, transient structural dynamic analysis was further introduced to rigorously examine the dynamic response performance, structural integrity, and underlying strain signal fidelity of the platform under realistic operating load conditions. Instead of using a simplified step load, this simulation mathematically fitted a vGRF curve based on experimental data from the literature and used it as the time-dependent load input for the transient analysis. This curve was derived from experimental data obtained by John J. McMahon using a force plate on a standard CMJ performed by an athlete with a BW of 67.5 kg and height of 180 cm [39], as shown in Figure 12. The entire jumping phase included the resting phase, squatting phase, propulsion phase, flight phase, and landing phase.

**Figure 13:**
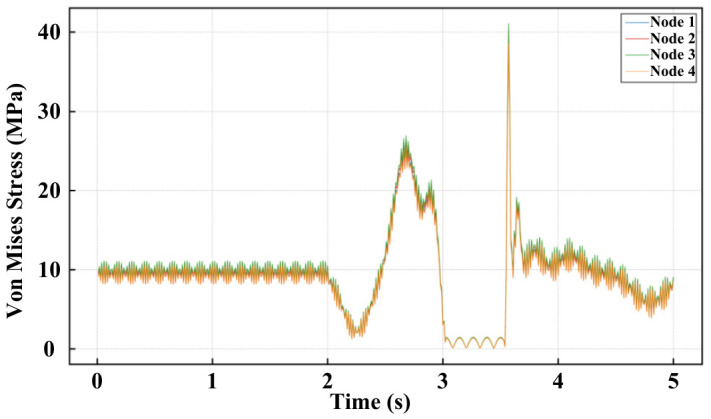
Dynamic stress variation patterns of multiple nodes within a single strain gauge attachment region.

**Figure 14:**
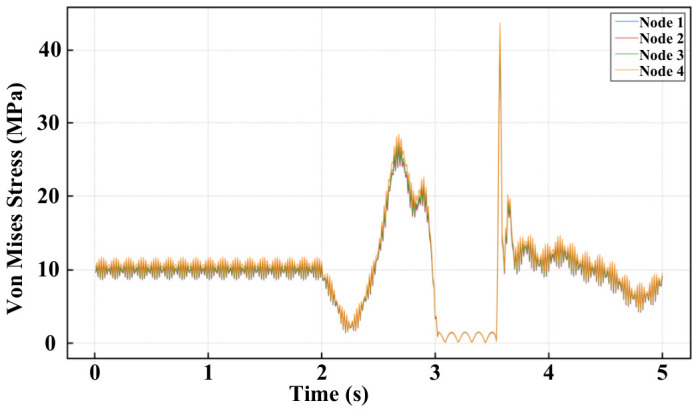
Dynamic stress variation patterns of nodes in the four strain gauge attachment regions on the same cantilever beam.

After the simulation, points in the four strain gauge attachment regions were randomly selected to analyze their time-stress dynamic responses. As shown in Figure 13, for different nodes within a single strain gauge attachment region, the equivalent stress variation patterns are highly consistent with each other and also consistent with the variation pattern of the fitted curve. As shown in Figure 14, for nodes in the four strain gauge attachment regions on the same cantilever beam, the equivalent stress variation patterns are almost identical, theoretically satisfying the stringent requirements of a full Wheatstone bridge circuit; the four curves all follow the same variation pattern as the simulated force curve.

### 3.2. Circuit Simulation

At the hardware design level, the DC linearity of the signal conditioning chain and the feasibility of the two-stage filtering architecture were demonstrated through static response and dynamic frequency domain simulations, respectively. During the pre-simulation phase of the full SPICE chain, the classic INA128 model (Texas Instruments, Dallas, TX, USA) was selected to theoretically validate the AC/DC transfer function and anti-aliasing filter topology of the AFE architecture. In the actual physical circuit, the core amplification stage was upgraded to a new-generation high-precision instrumentation amplifier INA828, which is pin-compatible with the simulated model. Compared with the simulation base model, the INA828 offers an ultra-low input offset voltage drift (0.5 μV/°C maximum) and an extremely high common-mode rejection ratio (CMRR > 120 dB), fundamentally improving the anti-interference capability and temperature drift suppression of the actual acquisition chain.

#### 3.2.1. Static Response

To construct an accurate full-bridge circuit simulation model, instead of using an ideal uniform stress, a discrete physical calibration over the 0–2000 N load range was performed using the actual structural deformations extracted from finite element simulations. Based on the standard piezoresistive principles, the local average stresses extracted from the FEA were converted into the absolute resistances of each bridge arm, as expressed in the following equation:

(4) $$\Delta R=\frac{R\cdot K\cdot \sigma }{E}.$$

**Figure 15:**
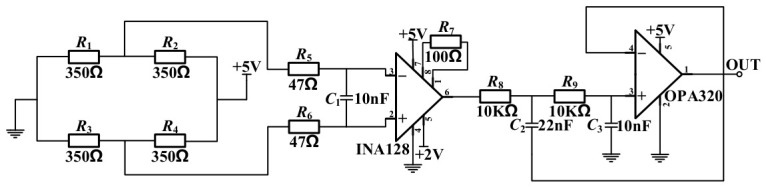
Static response simulation circuit diagram.

This non-ideal electromechanical co-simulation maximally reproduced the differential voltage output characteristics of the sensor under real full-load conditions. The simulation circuit and output data are shown in Figure 15 and Table 4, respectively.

**Figure 16:**
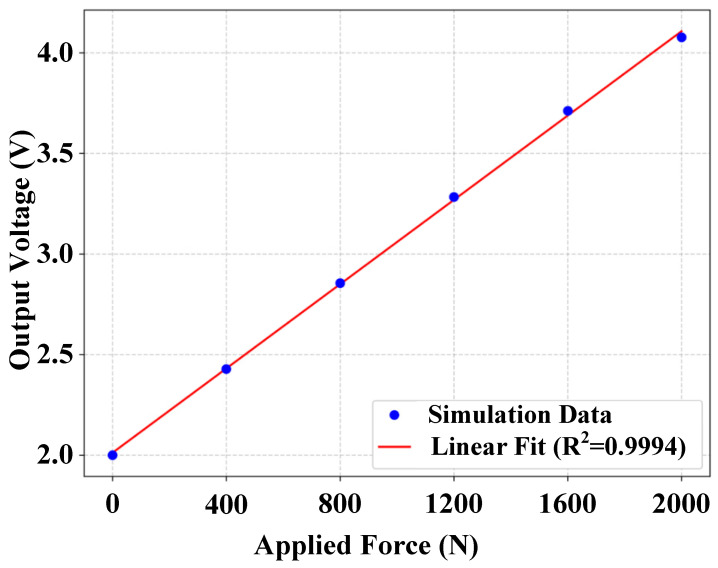
Static response and linear fitting curve of the circuit.

As shown in Figure 16, under non-ideal and asymmetric real physical deformation excitation, the system still exhibits good full-scale linear amplification capability ($R^{2}=0.9994$), verifying the high robustness and signal reconstruction fidelity of the hardware circuit in complex strain environments.

#### 3.2.2. Dynamic Frequency Domain

**Figure 17:**
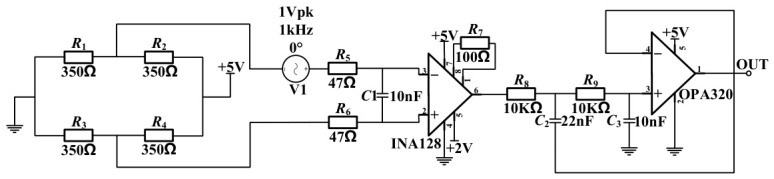
Dynamic frequency domain simulation circuit diagram.

The AC sweep method was adopted in the simulation to verify the frequency domain transfer characteristics of the cascaded filter network (the simulation circuit is shown in Figure 17).

**Figure 18:**
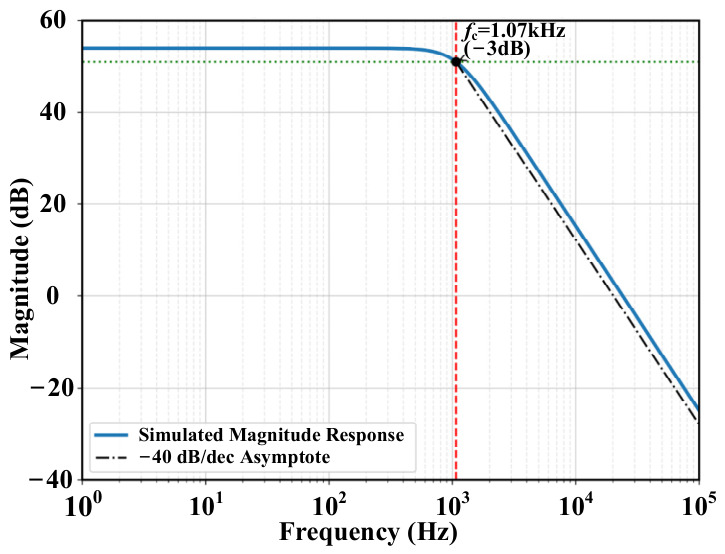
AC amplitude-frequency response and low-pass anti-aliasing analysis.

As shown in Figure 18, the system exhibits a stable Butterworth flat response in the low-frequency region (0–1 kHz), and the INA128 under high-gain configuration does not introduce parasitic attenuation to low-frequency signals, ensuring lossless amplification of the key spectral components of the vGRF during human jumping. After crossing the cutoff frequency (*f*_c_) of 1.07 kHz, the curve shows a significant second-order attenuation rate of −40 dB/dec, effectively suppressing mechanical high-frequency resonance and environmental power line/radio frequency interference, thereby providing an ideal anti-aliasing physical layer signal for the subsequent 16-bit analog-to-digital converter.

The circuit simulation results indicate that the proposed hardware topology is highly feasible in terms of impedance matching, range mapping, and anti-aliasing filtering, and can provide a high signal-to-noise ratio, distortion-free ideal physical layer input for subsequent high-speed 16-bit digital acquisition and microcontroller data processing.

## 4. Experimental Verification

### 4.1. Participants

Primary Validation Cohort: For the primary system validation, kinematic cross-validation, and test–retest reliability assessments, fourteen healthy young adults (seven males and seven females; age 23.7 ± 0.7 years; height 170.2 ± 7.7 cm; and body mass 62.0 ± 13.3 kg, range 42.9–85.0 kg) voluntarily participated in this study. Participants were recruited from Tianjin University of Science and Technology and represented a recreationally active young adult population. Inclusion criteria were: age between 18 and 30 years, no history of lower-limb musculoskeletal injury within the previous six months, and the ability to independently perform a CMJ without pain. Exclusion criteria included known vestibular or balance disorders, or participation in high-intensity lower-limb training within 48 h prior to testing.

Ecological Validity Cohort: Furthermore, to evaluate macroscopic dynamic accuracy through ecological validity comparisons with normative literature data, a validation cohort (*n* = six males; age 24.3 ± 0.5 years; height 177.5 ± 4.2 cm; body mass 80.2 ± 4.2 kg) was additionally recruited. This cohort was selected to provide anthropometric characteristics and loading conditions closer to those commonly reported in previous force-plate validation studies involving recreationally active adult populations. These participants followed the same inclusion and exclusion criteria as the primary cohort.

All participants provided written informed consent prior to testing. This study was approved by the Ethics Review of the Academic Committee of Tianjin University of Science and Technology (Approval No. TJKDLLSC2026041301), and all experimental procedures were conducted in accordance with the Declaration of Helsinki.

### 4.2. Experimental Setup and Movement Protocol for CMJ Testing

**Figure 19:**
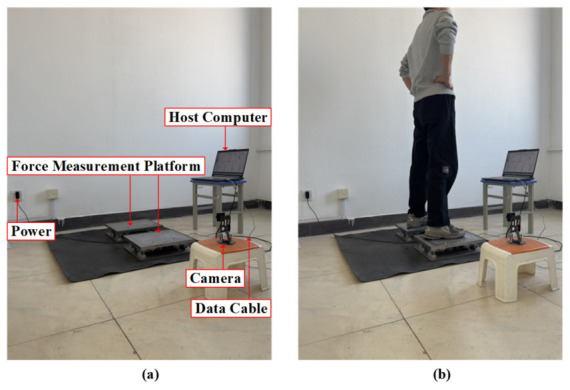
Schematic diagram of the experimental setup and standard CMJ testing protocol: (**a**) actual force plate testing setup; (**b**) initial quiet standing posture with hands on hips.

Before the formal test, the subjects completed a 5-min running warm-up and 3–5 practice jumps. During the formal test, the subjects stood with hands on hips and both feet placed on the geometric centers of the left and right force plates, respectively. This initial quiet standing posture was used to determine baseline BW. Subsequently, they performed maximal CMJs at a self-selected squat depth. They were required to maintain natural joint extension during takeoff and landing and to avoid knee tucking in the air as much as possible (as shown in Figure 19).

### 4.3. Statistical Analysis

Static and dynamic validations were performed using linear regression (reporting $R^{2}$ and root mean square error (RMSE)), MAPE, and Bland–Altman consistency analysis (reporting bias and 95% limits of agreement (LoAs)) [40]. The same methods were applied to the cross-validation of flight time.

For test–retest reliability across days, the relative reliability was assessed using the intraclass correlation coefficient (ICC) model (3, 1) [41] (interpretation criteria: <0.50 poor, 0.50–0.75 moderate, 0.75–0.90 good, >0.90 excellent), and the coefficient of variation (CV) was also reported. Absolute reliability was quantified using the standard error of measurement (SEM) and the 95% minimal detectable change (MDC_95_). Systematic bias was evaluated using a paired *t*-test and Cohen’s d effect size [42] (<0.20 trivial, 0.20–0.50 small, 0.50–0.80 moderate, >0.80 large).

Data exclusion criteria: A single jump was excluded if any of the following occurred: (1) both feet were not completely within the boundaries of their respective force plates; (2) the data integrity detection reported frame loss; and (3) the force curve exhibited signal abnormalities beyond physiologically reasonable ranges.

### 4.4. Static Physical Calibration and Sensitivity Coefficient Fitting

#### 4.4.1. Static Physical Calibration Experiment

Machining tolerances and AFE electrical offsets introduce inter-channel differences. Therefore, static physical calibration was performed on the eight independent acquisition channels using standard weights to extract an accurate electromechanical conversion matrix. The calibration procedure consisted of two stages:

- Center stepped loading: the load was increased stepwise from 8.587 kg to 78.104 kg to evaluate the linearity of the overall force plate structure and the symmetry of mechanical transmission.
- Single-channel independent loading: loads of 19.841 kg and 36.746 kg were applied directly above each sensor to suppress mechanical moment crosstalk and obtain the single-channel response sensitivity. The calibration data for the left and right plates are shown in Table 5 and Table 6, respectively.

**Figure 20:**
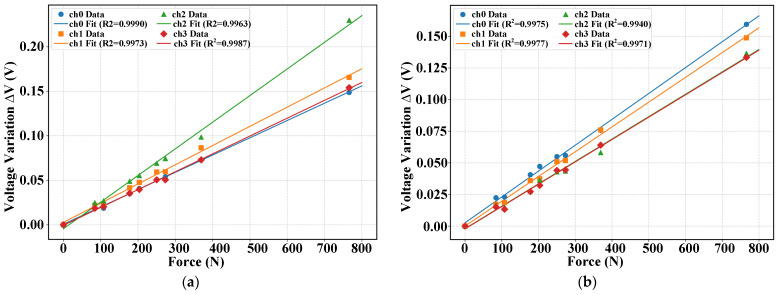
Static physical calibration response and linear fitting curves of the independent sensing channels of the bilateral force plates: (**a**) left force plate; (**b**) right force plate.

The test results (as shown in Figure 20) indicate that the system exhibits good static measurement performance and hardware chain stability. During the full-range loading process, all eight independent channels of the left and right force plates achieved an $R^{2}\geq 0.994$, and the response curves maintained good consistency near zero load. This result validates the linear amplification capability of the front-end AFE chain and supports the necessity of independently establishing the electromechanical conversion relationship for each sensing channel. At the same time, the system demonstrates stable mechanical recovery characteristics and electrical consistency as follows: taking the left plate as an example, after a full-range load-unload cycle, the maximum zero-voltage drift of each channel was 3.7 mV (corresponding to a baseline voltage range of 1.02–3.11 V). These results indicate that both hysteresis and zero drift of the system are controlled at low levels, meeting the requirements of high-precision real-time data acquisition for biomechanical measurements.

#### 4.4.2. Sensitivity Coefficient Fitting

The calibration data included both centers stepped loading and single-channel eccentric loading to ensure the column full rank of V and to fully excite the coupling characteristics between channels. Based on the measured data, the sensitivity vectors of the left and right force plates were obtained as follows:

(5) $$\left\{\begin{array}{l} K_{L}={\left[1270.21,904.84,824.89,1607.35\right]}^{T}(N/V)\\ K_{R}={\left[556.12,839.15,1872.05,2272.64\right]}^{T}(N/V) \end{array}\right..$$

### 4.5. Accuracy of Static Mass Measurement

Before the experiment, a calibrated commercial electronic scale (accuracy ± 0.1 kg) was used to measure the true BW of each subject as a reference. Each subject stood upright and motionless on the dual-plate force plate for at least 2 s. The host computer automatically recorded the resting vertical force and calculated the measured mass ($m=F_{\mathrm{static}}/g$). The mean of 10 measurements was taken as the system measurement value and used for correlation and consistency analysis with the reference values from the electronic scale. This design embedded the static accuracy assessment within the continuous dynamic testing, allowing simultaneous verification of the system’s baseline recovery stability after repeated impact loading.

**Figure 21:**
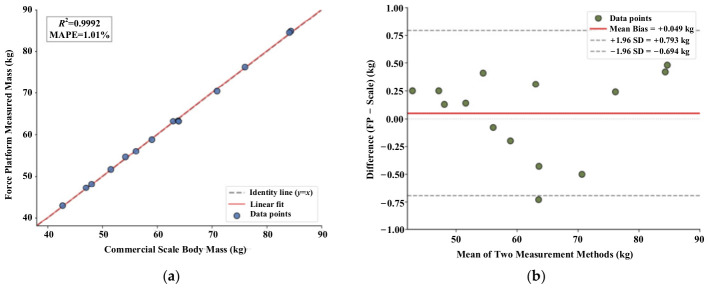
Accuracy and consistency analysis of human static mass measurement: (**a**) linear regression scatter plot of reference mass versus measured mass; (**b**) Bland–Altman plot.

The dual-plate force plate system demonstrated acceptable accuracy and stability during continuous in vivo static load measurements. As shown in Figure 21a, linear regression analysis revealed a high degree of agreement between the true reference mass and the mass measured by the force plate ($R^{2}=0.9992$). RMSE for in vivo measurements was 0.369 kg, and MAPE was effectively controlled at approximately 1.01%. Furthermore, the Bland–Altman analysis in Figure 21b further confirmed the reliability of the system under realistic testing conditions. The mean bias between the two measurement methods was extremely small (+0.049 kg), indicating that the system had no significant systematic overestimation or underestimation. The 95% LoA were narrow, spanning from −0.69 kg to +0.79 kg. Considering the inevitable minor body sway and postural noise during in vivo testing, this error range and agreement performance fully meet the requirements for high-fidelity biomechanical assessment.

To further evaluate the system’s sensitivity and accuracy under lower loading conditions, a low-load validation experiment was conducted. A series of calibrated weights (sandbags nominally at 5 kg, 10 kg, 20 kg, and 30 kg) were sequentially applied to the center of each force plate. Their exact masses (5.0 kg, 10.0 kg, 20.0 kg, and 30.0 kg) were pre-measured using the same commercial electronic scale (accuracy ± 0.1 kg) to serve as the standard reference. The static mass output from both the left and right plates was recorded and compared against the reference weights to assess the low-range linearity and measurement error.

**Figure 22:**
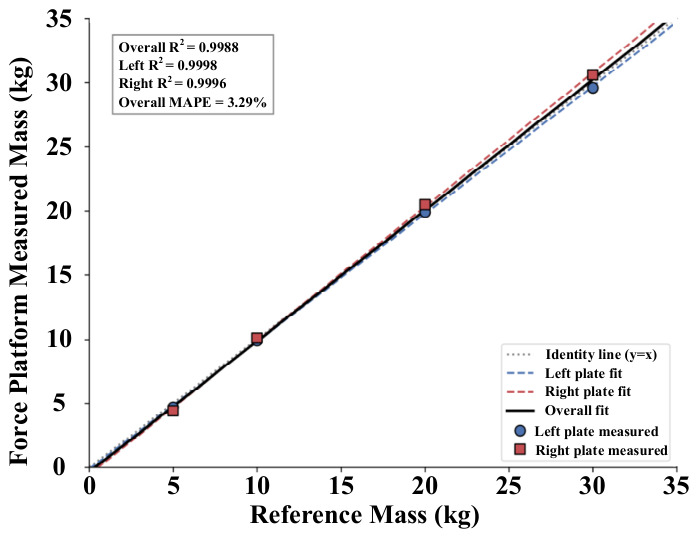
Linear regression analysis of the low-load static mass validation (5.0–30.0 kg).

The low-load validation experiment further confirmed the system’s sensitivity and acceptable linear tracking capabilities in lighter loading scenarios. As summarized in Table 7 and illustrated in Figure 22, across the smaller weight range of 5.0 kg to 30.0 kg, the system maintained a strong linear response ($R^{2}\geq 0.996$ for the combined bilateral dataset). The overall average absolute error across both plates was 0.34 kg, corresponding to a mean absolute percentage error (MAPE) of 3.29%. Notably, the left plate demonstrated exceptional accuracy (MAPE = 2.21%), while the right plate exhibited a slightly higher relative error (MAPE = 4.38%) mainly at the lowest load, which can be attributed to minor inherent variations in sensor pre-tension and analog gain. Overall, these realistic results demonstrate that the proposed force plate effectively captures subtle force variations, supporting its applicability for baseline posture measurements and partial-loading scenarios.

### 4.6. Accuracy and Linearity Analysis of Dynamic Impulse Measurement

This section used standardized physical loads to exclude human variability and independently evaluate the force–time integration accuracy of the system. Two calibrated dense sandbags (5 kg and 10 kg, exhibiting approximately inelastic collision upon ground contact) were dropped from five preset heights (h = 0.1, 0.2, 0.3, 0.4, 0.5 m) onto the geometric center of the force plate by horizontally withdrawing a support plate. Each mass–height combination was repeated three times on the left and right force plates, resulting in a total of 60 drops, with an interval of at least 10 s between consecutive drops. Assuming the sandbag is released from rest ($v_{0}=0\;m/s$) and undergoes a perfectly inelastic collision, the theoretical net impulse is calculated using classical kinematic formulas:

(6) $$I_{\mathrm{theory}}=m\sqrt{2gh}.$$

The measured impulse is extracted by integrating the net force from the 2000 Hz force–time curve:

(7) $$I_{\mathrm{mersured}}=\int_{t_{1}}^{t_{2}}(F(t)-mg)dt.$$

where $t_{1}$ and $t_{2}$ are the start and end instants of the impact event, respectively, automatically determined by the force signal first exceeding and finally returning to the static baseline threshold of ±5 N. It should be noted that the above theoretical impulse is based on the assumption of a perfectly inelastic collision. Actual sandbags may have a small coefficient of restitution; this potential source of error will be further analyzed in the Discussion (Section 5).

**Figure 23:**
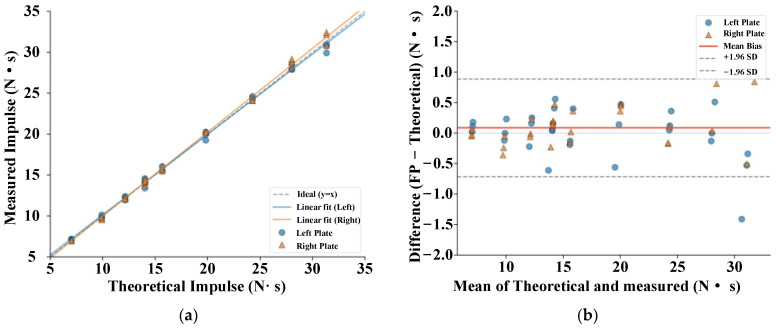
Accuracy and consistency analysis of dynamic impulse validation: (**a**) linear regression scatter plot of measured versus theoretical impulse; (**b**) Bland–Altman plot.

Figure 23 shows the linear regression and Bland–Altman agreement analyses between the theoretical impulse ($I_{\mathrm{theory}}$) and the hardware-measured impulse ($I_{\mathrm{measured}}$) over a wide dynamic range from 7.00 N·s to 31.32 N·s. The measured data from both the left and right force plates exhibited high agreement with the theoretical values.

The statistical results in Table 8 indicate that the $R^{2}$ for the two independent force plates reached high levels ($R^{2}>0.997$), demonstrating high linearity. Across all 60 drop tests, the absolute measurement error was well-controlled, with the MAPE strictly kept below 3%. Specifically, under the most extreme test condition (a 10 kg sandbag dropped from 0.5 m height generating a theoretical impulse of 31.32 N·s), the average measured impulses for the left and right plates were 30.56 N·s and 31.80 N·s, respectively. This confirms that the system maintains high measurement accuracy even under severe and instantaneous dynamic overload conditions.

### 4.7. Event Detection Accuracy and Time Synchronization

A 240 fps high-speed camera (frame interval ≈ 4.17 ms) was used solely for this validation experiment to establish an independent reference for flight time. The camera was positioned beside the force plate, with the lens horizontally aligned to the plate surface and the participant’s toe region. A total of 140 jumps were recorded. The video-based reference flight time ($\Delta T_{\mathrm{video}}=N/240$, where N is the number of frames between takeoff and landing) was obtained through manual frame-by-frame inspection using the open-source video analysis software Kinovea (version 0.9.5).

To ensure consistency, the operational criteria for key biomechanical events were explicitly defined. The takeoff event was identified as the first video frame in which the participant’s toes completely lost physical contact with the force plate surface, whereas the landing event was defined as the first frame in which any part of the foot re-established contact with the plate. All video annotations were performed by a single experienced analyst. To evaluate intra-rater reliability, 20% of the video trials (*n* = 28) were randomly selected and re-analyzed blindly by the same analyst after an interval of approximately 8 weeks. Reliability was assessed using a two-way mixed-effects, absolute-agreement, single-rater ICC. The analysis yielded an excellent ICC of 0.996 (95% CI: 0.988–0.999) for the manually identified event frame indices, confirming the robustness and repeatability of the manual event identification procedure.

The flight time measured by the force plate was automatically extracted using an adaptive dynamic threshold algorithm. The event detection threshold was set to 2.5% of the subject’s resting BW or 15 N, whichever was larger. When the filtered vertical resultant force remained continuously below this threshold for more than 30 ms, the takeoff instant was identified. When the force rose back above the threshold and remained so for 50 ms consecutively, the landing instant was identified. This asymmetric consecutive-frame window design (30 ms for takeoff, 50 ms for landing) was specifically intended to suppress misjudgments caused by mechanical oscillations during takeoff unloading and landing impact. The two sets of flight times obtained from video and force plate were then used for correlation and Bland–Altman consistency analysis.

**Figure 24:**
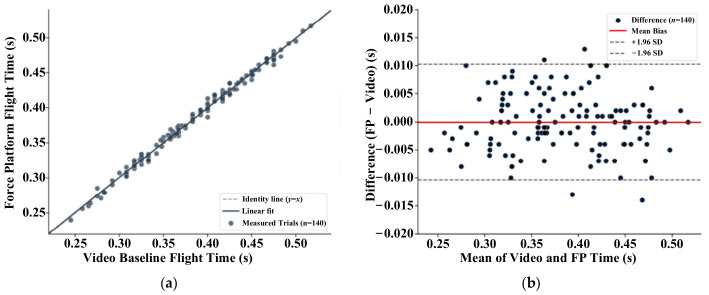
Cross-validation of flight time between the force plate and high-speed video: (**a**) linear regression scatter plot of video-based reference time versus force-plate-measured time; (**b**) Bland–Altman plot.

The force plate system demonstrated high consistency with the 240 fps high-speed camera in detecting takeoff and landing events. As shown in the scatter regression plot of Figure 24a, the force-plate-measured flight times and the video-based reference times for the 140 valid jumps exhibited a high linear correlation ($R^{2}=0.9929$), with all data points tightly concentrated near the y=x line and an RMSE of only 0.0052 s. Furthermore, the Bland–Altman analysis in Figure 24b confirmed that the force plate system did not suffer from any significant time-axis delay or premature triggering. The mean bias between the two methods was extremely small (−0.0001 s), and the 95% LoA were tightly controlled between −0.010 s and +0.010 s.

### 4.8. Synchronization Validation of Force–Time Curves and Kinematic Features

**Figure 25:**
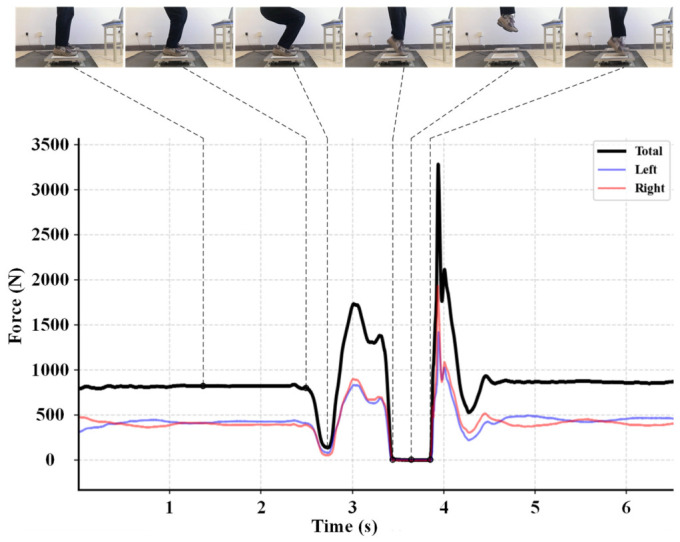
Demonstration of cross-modal time synchronization and jump phase identification.

To demonstrate the time synchronization accuracy of the system at the application level, Figure 25 presents the cross-modal synchronization results of the force–time curve and the subject’s sagittal plane kinematic features. The upper part shows key kinematic video frames synchronously captured by the hardware trigger (including initial resting, movement onset, lowest squat position, takeoff, flight, and landing with absorption); the lower part shows the strictly time-aligned synchronized filtered force–time curve. The dashed lines indicate the core biomechanical events extracted by the adaptive threshold algorithm, validating the stable synchronization performance of the force signal in movement segmentation.

Based on the unified time reference established by the hardware trigger mechanism, the host computer software can accurately match the underlying kinetic features with the video frames. As indicated by the dashed lines in Figure 25, key biomechanical events derived from the force signals, such as the lowest squat position and the takeoff instant, achieve perfect visual correspondence with the subject’s actual movement trajectory captured by video. This result intuitively demonstrates that the proposed adaptive threshold algorithm not only accurately captures extreme points at the mathematical level, but also that its segmentation results possess high physical authenticity, effectively eliminating the non-deterministic time delay errors commonly encountered in multi-system integration.

### 4.9. Test–Retest Reliability of Core Kinetic Variables

Fourteen subjects completed the test–retest across two testing sessions (Session 1 and Session 2) separated by 48 h. Both sessions were conducted in the same laboratory at a room temperature of 22–25 °C, with testing time uniformly scheduled between 9:00 AM and 12:00 PM. In each session, the subjects performed the same warm-up followed by five maximal CMJs (movement protocol as described in Section 4.2), with a 60 s rest interval between jumps.

The host computer system automatically extracted the following five core variables: concentric impulse, jump height, PF, rate of force development at 100 ms (RFD_100ms_), and ASI. The definitions of these variables are provided in Section 2.4.2. For each subject, the mean values of the five jumps from each session were used for paired analysis (detailed statistical results are presented in Table 9).

**Figure 26:**
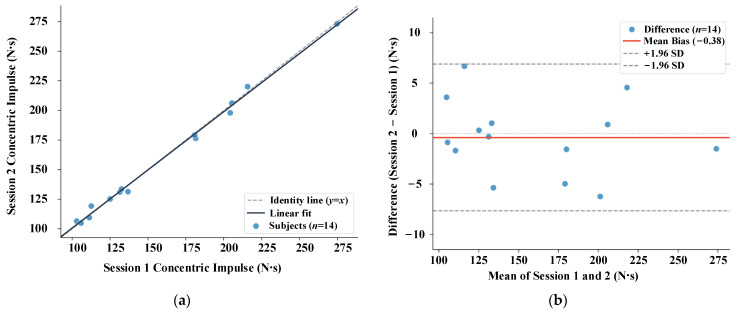
Test–retest reliability analysis of concentric impulse between the two testing sessions: (**a**) linear regression scatter plot of mean impulse between Session 1 and Session 2; (**b**) Bland–Altman plot.

As shown in Table 9, the dual-channel force plate system demonstrated good to excellent measurement consistency in the test–retest validation across day. As the core reflection of the system’s underlying force measurement accuracy, concentric impulse exhibited the highest test–retest reliability among all variables (ICC = 0.997, CV = 1.30%). Jump height, derived from the impulse-momentum relationship, also showed high stability (ICC = 0.987, CV = 3.04%). The scatter plot and Bland–Altman plot in Figure 26 further confirmed these results: the data points were highly concentrated around the line of identity, with a mean bias of only −0.18 N·s and extremely narrow 95% LoA spanning from −7.59 to 7.22 N·s. These findings confirm the absence of systematic measurement drift between the testing sessions.

**Figure 27:**
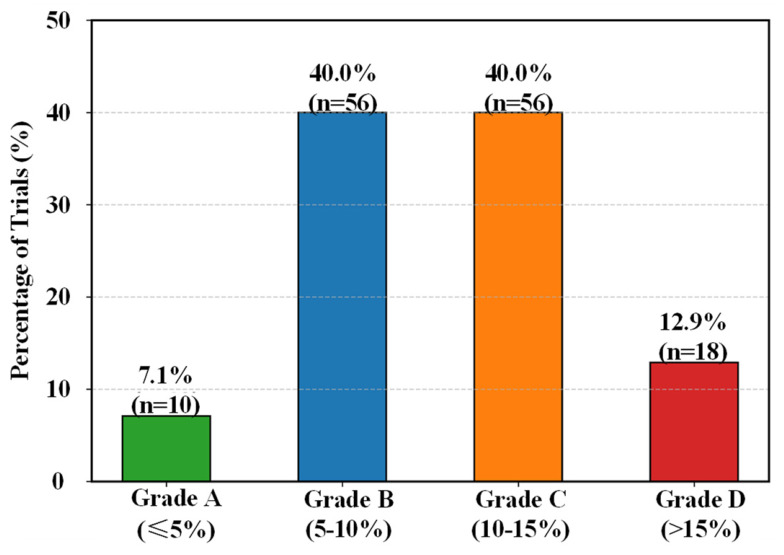
Quality rating distribution for the cross-validation of the flight time method and the impulse-momentum method.

Regarding the transient kinetic variables, PF maintained a high level of reliability (ICC = 0.962, CV = 4.63%). In addition, RFD_100ms_ also achieved a good reliability level (ICC = 0.883, CV = 10.32%). ASI, calculated based on the independent PF values, exhibited the highest within-subject variability among all variables (ICC = 0.748, CV = 21.78%), consistent with the high variability characteristic of this metric as a ratio of two instantaneous extreme values. Paired *t*-tests showed no significant differences for any variable between the two sessions (*p* > 0.35), and Cohen’s d analysis further confirmed the absence of substantial systematic bias (|*d*| ≤ 0.24). Furthermore, the distribution of cross-validation quality ratings for the 140 jumps using multiple physical laws was as follows: Grade A accounted for 7.1% (10 jumps), Grades B and C each accounted for 40%, and 12.9% (18 jumps) exhibited an inter-algorithm discrepancy greater than 15% (Grade D). The vast majority of trials (87.1%, *n* = 122) fell within the acceptable range of Grades A to C (relative deviation ≤ 15%), with only a small proportion (12.9%, *n* = 18) rated as Grade D (>15%), as shown in Figure 27. This distribution indicates that the two jump height estimation methods maintained acceptable overall consistency in the vast majority of trials, and this result was also used to support the rationale for the system’s default adoption of the impulse-momentum method.

### 4.10. Ecological Validity Comparison with Published Commercial Force-Plate Data

A cross-sectional ecological validation experiment was conducted using an additional validation cohort (*n* = 6) to compare the macroscopic kinetic outputs of the proposed system with published commercial force-plate reference data. Testing was performed under laboratory conditions and movement protocols closely matched to those described in the reference AMTI-based literature [43].

Prior to testing, all participants completed a standardized warm-up (~10 min) consisting of dynamic stretching and submaximal CMJs to reduce injury risk and familiarize participants with the movement task. The formal testing protocol consisted of three maximal CMJs performed with approximately 60 s inter-jump rest intervals. Participants were instructed to jump as fast and as high as possible while maintaining the prescribed upper-limb posture and keeping both feet on their respective force plates throughout the movement.

Notably, although the proposed dual-plate system was originally designed to support force acquisition at 2000 Hz, the sampling frequency was intentionally configured to 1000 Hz during this experiment to better align with the acquisition settings commonly reported in commercial AMTI-based force-plate studies. The movement protocol and signal-processing workflow were likewise matched as closely as possible to the reference literature.

The proposed dual-plate system automatically extracted the same core kinetic variables used in the primary validation analysis, including jump height, flight time, take-off velocity, concentric impulse, and PF. For each participant, the mean value of the three valid jumps was used for subsequent analysis.

Ecological validity was assessed by comparing the group-level mean and standard deviation of the present cohort with published reference values obtained from commercial force-plate studies using AMTI systems under comparable CMJ conditions [43]. This comparison aimed to determine whether the macroscopic outputs of the proposed system fell within the biomechanically expected range reported in the literature, rather than to provide a direct synchronous criterion-validation against a commercial force plate. Because the reference values were derived from the published literature rather than simultaneously recorded commercial-plate measurements, the present analysis should be interpreted as an ecological validity comparison. Relative percentage differences between the present system and the published AMTI reference values were additionally calculated for descriptive comparison.

As shown in Table 10, the descriptive statistics indicate that the kinetic metrics generated by our custom system are broadly consistent with the AMTI reference data. The small proportional differences in absolute impulse and PF may be partially explained by the slightly lower mean body mass of the present cohort relative to the reference group. Although this cross-sectional comparison does not establish direct concurrent validity, it supports the macroscopic ecological validity of the custom dual-plate system and suggests that its outputs fall within the expected physiological and biomechanical range for adult CMJ testing.

## 5. Discussion

### 5.1. System Underlying Accuracy and Dynamic Response

Static accuracy and dynamic response capability are the foundations for performing complex kinetic analyses in biomechanical measurement systems. The results of this study show that the proposed dual-channel force plate achieves acceptable accuracy in in vivo static mass measurement (MAPE = 1.01%, $R^{2}=0.9992$), and the Bland–Altman analysis reveals a very small mean bias (+0.049 kg) and narrow LoA (−0.69 to +0.79 kg), indicating that the system has good static measurement consistency under the in-vivo testing conditions of this study. Combined with the dual-plate structural design, these results suggest, to some extent, that the assembled structure does not introduce significant mechanical transmission loss or lateral crosstalk. Furthermore, the low-load static validation confirmed that this linear measurement capability ($R^{2}\geq 0.996$) extends to lighter mass ranges (5.0–30.0 kg). While a slightly elevated relative error was observed at the lowest 5.0 kg load (attributable to inherent sensor pre-tension variations and analog noise floors), the overall absolute error remained minimal (0.34 kg). This indicates that the system possesses adequate sensitivity for baseline static posture assessments and partial-loading applications, effectively complementing the full-range in vivo results.

In the free-fall dynamic validation, the system exhibited a high linear relationship ($R^{2}>0.997$) over the impulse range of 7.00 to 31.32 N·s, with a MAPE below 3%. The Bland–Altman analysis showed a slight systematic underestimation of the measured impulse compared to the theoretical value. This can be mainly attributed to the following two unavoidable physical losses: first, the internal deformation of the sandbag upon ground impact is accompanied by a limited coefficient of restitution, causing a small amount of momentum not to be fully converted into vertical impulse; second, the instantaneous withdrawal of the support plate may introduce a slight initial velocity deviation or frictional damping. Despite these losses, the system exhibited stable force–time integration capability under high-magnitude transient impacts.

It is worth noting that the static mass measurements were obtained during the intervals between consecutive CMJ tests, while a single jump can generate a transient PF exceeding 2000 N. Visual inspection did not reveal any obvious baseline shift in the system before and after the continuous jumps. However, considering the minor relative measurement deviations observed during the 5.0 kg low-load testing, it is clear that hardware noise floors and minor zero-drifts can still affect sensitivity at the extreme low end of the measurement range. Therefore, although this study did not perform a systematic quantitative assessment of zero drift, the long-term fatigue performance and drift characteristics of the system still require further validation.

### 5.2. Consistency Verification Between Simulation Predictions and Experimental Results

To evaluate the self-consistency of the entire “design → simulation → physical prototype” chain, this study compared the predictions from FEA and circuit simulations with the measured data. In terms of static sensitivity, the stresses predicted by FEA in each sensing region exhibited a high linear relationship with the external load ($R^{2}\geq 0.9999$), and the voltage–load response of each channel in the actual calibration also showed excellent linearity ($R^{2}\geq 0.999$). The consistency in the magnitude of linearity between the two supports, to some extent, the validity of the electromechanical conversion chain from the stress field to the bridge output. Furthermore, based on standard piezoresistive principles and the configured amplifier gain, the stress values predicted by the FEA were theoretically converted into amplified voltage outputs. Taking a 400 N load applied to the right plate as an example, the FEA-predicted theoretical output voltage for channel ch2 was 2.428 V, while the measured calibration data showed an output voltage of 2.596 V under the same load, giving a deviation of approximately 6.92%. This magnitude of deviation can be mainly attributed to machining tolerances, transmission loss in the strain gauge adhesive layer, and the actual tolerance of the gain resistor, indicating that the electromechanical co-simulation model possesses a certain predictive capability.

In terms of dynamic characteristics, the FEA predicted a first natural frequency of approximately 206 Hz for the system, which is higher than the main frequency range of human lower limb force production (<100 Hz) [44], suggesting that the risk of structural resonance under normal operating conditions is low. Meanwhile, the cutoff frequency of the second-order Sallen–Key low-pass filter on the hardware side was designed to be approximately 1.07 kHz, providing a certain degree of attenuation of potential high-frequency oscillations while preserving the target biomechanical signals. A mutually supportive trend was observed among the FEA modal predictions, the hardware filter design, and the stability of the measured signals.

At the signal chain level, the circuit simulation showed that the AFE exhibited good linear amplification characteristics under DC conditions ($R^{2}\geq 0.999$), and the AC sweep simulation confirmed the flat response in the low-frequency region and the second-order attenuation characteristic after the cutoff frequency. The measured calibration data also showed stable linear responses for each channel, further supporting the feasibility of the simulation-adopted topology and component parameter selection in the physical implementation.

Taken together, the FEA, circuit simulation, and experimental measurements showed consistent trends in static linearity, dynamic frequency characteristics, and signal chain gain, supporting to some extent the development process of “simulation guiding design, experiment validating simulation” adopted in this study.

### 5.3. Validity of Flight Time Measurement

Accurate measurement of flight time depends on precise identification of takeoff and landing events and is therefore influenced not only by the hardware sampling rate but also by the anti-interference capability of the threshold algorithm against baseline fluctuations. In this study, the flight time measured by the force plate was compared with the frame-by-frame analysis results from a 240 fps high-speed video. The LoA between the two methods were found to be approximately ±10 ms, which is on the order of the temporal resolution error of the video system (approximately ±4.16 ms). This quantitative result echoes the qualitative observation of cross-modal visual alignment (Figure 25), further indicating that under the current sampling rate, filter settings, and threshold determination conditions, the force-threshold-based event detection algorithm can reliably identify takeoff and landing instants, with no obvious systematic advance or lag observed. These results are comparable to the flight time validity levels reported for commercial force plates in related studies [45,46], supporting the applicability of the event detection logic of the proposed system in CMJ testing.

### 5.4. Test–Retest Reliability of Core Kinetic Variables Across Days

Before discussing the reliability of each core variable individually, a global methodological factor that affects the interpretation of all ICC values should be noted first. This study included a mixed-sex sample with a wide range of BWs (42.9 to 85.0 kg). According to the statistical definition of the ICC, this high heterogeneity increases the true between-subject variance and may uniformly inflate the ICC values for all kinetic variables in this system [41]. Therefore, when evaluating across studies, the CV, as an absolute consistency metric not affected by between-group variance, should be used together with the ICC to provide a more comprehensive reliability assessment. The discussion of each variable below is conducted under this premise:

- Concentric impulse and jump height—Concentric impulse showed extremely high test–retest reliability (ICC = 0.997, CV = 1.30%), with a CV value falling within or below the range reported for some commercial force-plate systems in previous studies [47,48]. This is mainly attributable to the natural smoothing effect of integration on high-frequency noise. Jump height derived from the impulse also exhibited excellent stability (ICC = 0.987, CV = 3.04%), which is generally consistent with the reliability levels reported for modern commercial force plates [48,49]. In addition, the Bland–Altman analysis showed very small mean biases (−0.18 N·s and −0.17 cm), confirming that the system has no significant systematic shift between the two testing sessions. It is noteworthy that in the cross-validation of jump height using multiple physical laws, the vast majority of trials (87.1%) had relative deviations distributed within the acceptable Grades A to C, with only 12.9% falling into Grade D. This not only demonstrates that the system did not suffer from integration drift or frame synchronization loss at the underlying level, but also that the phenomenon where the moderate deviations were concentrated in Grade B (40%) and Grade C (40%) is fully consistent with biomechanical expectations: the postural compensations (such as hip and knee flexion) commonly observed during landing buffering physically prolong the true flight time, inevitably leading to a systematic and reasonable overestimation by the flight time method [45]. Therefore, this distribution not only rules out instrumentation error and objectively reflects the mechanical characteristics of human joints, but further supports the scientific soundness of the system’s design decision to adopt the impulse-momentum method, which is more robust against posture interference, as the standard kinetic output;
- PF—The reliability of PF was slightly lower than that of impulse and jump height, but still at a high level (ICC = 0.962, CV = 4.63%), which is consistent with the reliability ranges reported for commercial devices in the existing literature [47,48]. This difference may be related to the signal characteristics of this variable: PF reflects an instantaneous extreme value on the force-time curve; compared with the integrated quantity over the entire phase, it is not only more susceptible to small variations in individual force production rhythm, but also more sensitive to the data smoothing and sampling accuracy of the underlying algorithm [50]. Nevertheless, its high ICC and the paired *t*-test result showing no significant systematic difference (*p* = 0.380) indicate that the 2000 Hz sampling rate provides sufficient temporal resolution for the stable capture of transient extreme values;
- RFD—RFD_100ms_ exhibited good test–retest reliability (ICC = 0.883, CV = 10.32%). As a derived variable calculated from force changes within a short time window, RFD is sensitive to the determination of the onset of force production, signal noise level, and filtering processing; therefore, its reliability is generally lower than that of integrated variables such as impulse and PF [50]. The results of this study are consistent with this general trend. In terms of absolute consistency, the CV value of RFD_100ms_ in this study (10.32%) is at or better than the lower bound of the variation range of similar derived variables reported in recent studies using commercial force plates (e.g., Anicic et al. [51] reported CV ≥ 22.2% for kinetic derived variables; Godhe et al. [47] reported CV values for RFD ranging from 12.9% to 54.6% on a portable commercial force plate), suggesting that the proposed system exhibits acceptable measurement stability for capturing high-frequency transient kinetic features;
- ASI—ASI calculated based on the independent PF values exhibited the highest within-subject variability among all variables (ICC = 0.748, CV = 21.78%). This result is consistent with the recognized high variability of asymmetry metrics in sports biomechanics. As pointed out by Bishop et al. [52] in their methodological guidelines, because the sample mean of asymmetry metrics such as ASI often approaches zero, small unilateral mechanical fluctuations can mathematically inflate the CV substantially; therefore, the reliability assessment of such metrics typically relies more heavily on ICC values. Previous empirical studies have likewise reported relatively low reliability for asymmetry metrics. For example, Pérez-Castilla et al. [53] reported ICC values ranging from only 0.15 to 0.64 for kinetic asymmetry variables during CMJs.

Although the ASI in the present study demonstrated moderate relative reliability (ICC = 0.748) compared with the previous literature, its absolute stability remains limited, as reflected by the relatively high CV. This performance may partially benefit from the completely physically isolated dual-plate architecture, which reduces the mechanical crosstalk inherent in traditional single-plate systems during bilateral force separation. Nevertheless, the high variability of ASI likely reflects the inherent biological variability of human neuromuscular control rather than solely hardware-related measurement uncertainty. During repeated jumping tasks, subjects frequently exhibit subtle subconscious weight-shifting strategies and motor-control compensations between the left and right lower limbs. Because subjects rarely maintain stable dominance of the same lower limb across consecutive jumps [53], this inconsistent switching of the dominant side likely contributes substantially to the inherent variability of ASI measurements.

Consequently, the practical application of ASI should be interpreted cautiously. The elevated CV suggests a relatively large MDC, thereby limiting the sensitivity of ASI for detecting subtle individual longitudinal changes. Therefore, when utilizing this system for routine training and performance monitoring, practitioners should avoid overinterpreting isolated single-trial ASI values. Instead, it is recommended to use averaged ASI values derived from multiple valid trials and to interpret asymmetry findings within the broader context of the subject’s overall biomechanical performance.

### 5.5. Hierarchical Pattern and Comprehensive Evaluation of Kinetic Variable Reliability

Taken together, the coefficients of variation for the variables in this study present the following clear hierarchical pattern: concentric impulse (1.30%) < jump height (3.04%) < PF (4.63%) < RFD_100ms_ (10.32%) < ASI (21.78%). This result is generally consistent with the reliability hierarchy of force-plate kinetic variables identified by Huebner et al. using generalizability theory (G-Theory) [54]: integrated and macroscopic performance variables (e.g., impulse, height) exhibit the highest single-test stability, followed by transient extreme values (PFs), while derivative-type (RFD) and ratio-type (ASI) variables show greater variability due to their high sensitivity to physical boundary noise. This hierarchical relationship suggests that the reliability differences among kinetic variables may primarily originate from their inherent biomechanical properties and mathematical extraction algorithm characteristics, rather than from pure instrumentation hardware noise.

From a practical perspective, the MDC_95_ values reported in this study may provide preliminary reference thresholds for sport performance monitoring in healthy CMJ testing contexts. For example, when concentric impulse changes by more than 7.26 N·s or jump height changes by more than 1.31 cm, the observed difference may more likely reflect a genuine performance alteration rather than measurement error.

### 5.6. Ecological Validity Relative to Published Commercial Force-Plate Data

The cross-sectional comparison with published AMTI-based force-plate data offers an additional ecological reference for evaluating the proposed system. Under a closely matched CMJ protocol and a deliberately harmonized 1000 Hz sampling configuration, the proposed dual-plate system yielded jump height, flight time, take-off velocity, concentric impulse, and PF values (Table 10) that were broadly comparable to the ranges reported in the commercial force-plate literature. These findings suggest that the macroscopic kinetic outputs of the proposed system fall within the expected physiological and biomechanical range for adult CMJ testing.

Nevertheless, because the comparison was based on published reference data rather than simultaneous measurements obtained from a commercial force plate under identical experimental conditions, the present analysis should be interpreted as supportive ecological-validity evidence rather than formal concurrent criterion validation. Direct agreement analyses (e.g., Bland–Altman analysis and synchronous criterion-validation against simultaneously collected commercial force-plate measurements) remain necessary in future work to establish rigorous cross-platform concurrent validity.

### 5.7. Limitations

This study has the following limitations. First, the sample size was small (*n* = 14), which, although meeting the basic requirements for reliability studies [55], employed a mixed-sex design that increased the true between-subject variance and may have inflated the ICC values; therefore, the relevant metrics and their relatively wide confidence intervals should be interpreted with caution. Second, in terms of application scenarios and external validity, the current evaluation was conducted only for the single task of CMJ within a controlled laboratory setting. As an intentional methodological choice, this study focused exclusively on CMJ analysis to prioritize the validation of the proposed acquisition and synchronization pipeline. Although an ecological-validity comparison with published AMTI-based force-plate data was additionally performed, a fully synchronized simultaneous comparison with a commercial force plate under identical experimental conditions was still lacking. Therefore, the applicability of the system in complex field environments (e.g., temperature fluctuations) and in other testing paradigms (e.g., landing deceleration, isometric testing), as well as its strict cross-platform consistency, remain to be further examined. Third, the cutoff frequency of the AFE filter in this study was set close to the Nyquist frequency; its design goal was primarily to suppress high-frequency noise rather than to completely eliminate aliasing effects. Considering that the spectral characteristics of human movement signals are mainly concentrated in the low-frequency region (typically below 100 Hz), this study further implemented band limitation via back-end digital low-pass filtering, thereby effectively reducing the potential impact of aliasing in practical applications. Finally, with regard to system calibration and quantitative validation, the present study mainly focused on vertical kinetic characteristics. A rigorous two-dimensional position calibration experiment, in which known loads are applied at predefined grid locations to quantify CoP error, has not yet been performed. Therefore, the current CoP output should be regarded as an auxiliary qualitative indicator for stance centering and data quality monitoring rather than a validated quantitative measure for posturography or fine balance assessment. In addition, the comparison between simulation and measurement remains qualitative at this stage.

### 5.8. Open Architecture, Cost-Effectiveness, and Future Research Directions

To address the closed nature of commercial devices at the underlying algorithm level, the primary engineering contribution of this system is to provide a fully transparent open-architecture solution. Researchers can not only obtain the underlying hardware triggering logic but also flexibly adjust core settings such as the baseline calculation strategy, filter parameters, and integration time window in the host computer. This architecture design, which enhances system transparency, gives researchers the freedom to break through the constraints of closed ecosystems and adapt to the specific requirements of different complex research scenarios.

Based on this transparent architecture, the system further demonstrates high engineering accessibility. While maintaining the overall measurement consistency and reliability levels described above, the estimated bill-of-material cost of the system is strictly controlled below 500 USD (custom structural components approximately 250 USD, with the remaining costs approximately 200 USD). Compared with mainstream commercial devices priced above 15,000 USD, the proposed system exhibits comparable macroscopic CMJ kinetic assessment performance under the present validation conditions at a hardware cost of less than 5% of the latter. This extremely high cost-effectiveness serves as a key support and is expected to significantly improve the accessibility of multimodal biomechanical measurement equipment in budget-constrained laboratories and grassroots sports performance settings.

Future research can be further extended in the following directions. First, the reliability of the system should be validated in larger and more homogeneous populations, and its sensitivity to changes induced by training interventions should be evaluated. Second, rigorous two-dimensional positional calibration experiments using known loads applied at predefined grid locations, together with eccentric loading validation and measured frequency-response characterization, should be performed to establish a more comprehensive quantitative validation framework for both CoP estimation and dynamic force measurement across a wider range of spatial loading conditions. Third, the influence of different environmental conditions and long-term usage on system stability should be analyzed. Fourth, although the present study included an ecological-validity comparison against published commercial force-plate data, future studies should further conduct fully synchronized simultaneous comparisons with commercial force plates to establish rigorous cross-platform concurrent validity evidence. Fifth, building upon the current algorithmic framework, future work will extend the open-source system to other jump modalities, including SJ and DJ. Finally, taking advantage of the open architecture of the system, multimodal data fusion studies with electromyography and optical motion capture systems can be pursued within an open-source framework.

## 6. Conclusions

This study designed, developed, and evaluated a dual-channel strain gauge force plate system featuring an open architecture and hardware-level synchronized multimodal acquisition. The system integrates a physically isolated dual-plate mechanical structure, a hardware-level video trigger synchronization mechanism, and a customized adaptive analysis algorithm, enabling stable extraction of full-phase kinetics and cross-modal kinematic features during CMJs. The main contributions and core findings are summarized as follows:

1. Underlying measurement performance and ecological validity: In in vivo static mass measurements, the system demonstrated high precision and consistency (MAPE = 1.01%, R^2^ = 0.9992, Bland–Altman mean bias = +0.049 kg). Crucially, low-load validation confirmed that this highly linear measurement capability (R^2^ ≥ 0.996) is effectively maintained even at lighter loading conditions (5.0–30.0 kg). Free-fall dynamic impulse validation showed excellent full-scale linear response (R^2^ > 0.997), and no obvious zero drift was observed after repeated high-amplitude dynamic loading. FEA and circuit simulations further supported the linearity and dynamic characteristics of the system from the aspects of structural mechanics and signal conditioning. Furthermore, the comparison with published AMTI-based force-plate data suggests that the macroscopic kinetic outputs of the proposed system fall within the expected physiological and biomechanical range for adult CMJ testing.
2. Cross-modal time synchronization performance: Benefiting from the underlying hardware trigger and data frame binding strategy, the LoA for flight time between the system and a 240 fps high-speed camera was strictly controlled within ±10 ms. This not only verifies the absence of underlying time drift during multi-system integration but also confirms the high physical fidelity of the adaptive force-threshold algorithm in jump event segmentation from a cross-modal visual perspective.
3. Test–retest reliability of core kinetic variables: In the across-day in vivo retest, macroscopic integrated variables demonstrated very high test–retest stability (concentric impulse: ICC = 0.997; jump height: ICC = 0.987), while transient peak variables (PF and RFD_100ms_) also reached good reliability levels. The physically isolated dual-plate architecture effectively eliminates the mechanical crosstalk inherent in traditional single-plate systems, thereby enabling independent bilateral lower-limb force assessment. However, given the only moderate reliability and relatively high biological variability of the ASI (ICC = 0.748, CV = 21.78%), this asymmetry metric should be interpreted cautiously in longitudinal individual monitoring.
4. System transparency, customizability, and cost-effectiveness: Unlike the closed-algorithm nature of traditional commercial systems, this system provides a fully transparent data analysis architecture. Researchers can directly access and adjust core parameters such as baseline calculation, filter cutoff frequency, and integration time window, significantly enhancing data processing flexibility and controllability. On this architectural basis, the hardware BOM cost is below 500 USD, which is less than 5% of the price of mainstream commercial equipment, greatly lowering the implementation threshold for multimodal biomechanical measurement systems.

In summary, this work not only provides a stable, anti-interference multimodal testing system for sports biomechanics, but also breaks down the algorithmic barriers of traditional commercial instruments through an open-source transparent architecture and cost-effective hardware implementation. The MDC_95_ values reported in this study may provide preliminary reference values for general training monitoring in healthy populations. The proposed system holds substantial potential for widespread application in grassroots sports performance monitoring and large-scale multimodal fusion research.

## Figures and Tables

**Table 1:** Mechanical properties of materials in the finite element model.

Material	Elastic Modulus (MPa)	Poisson’s Ratio	Yield Strength (MPa)
Structural steel	2.05 × 10^5^	0.29	530
Aluminum alloy	7.4 × 10^4^	0.33	317

**Table 2:** Average stress results of each sensing region on a single beam under different center loads.

Force (N)	Top Strain Gauge 1 Average Stress (MPa)	Top Strain Gauge 2 Average Stress (MPa)	Bottom Strain Gauge 1 Average Stress (MPa)	Bottom Strain Gauge 2 Average Stress (MPa)
0	0	0	0	0
400	6.36	6.33	6.38	6.23
800	12.72	12.67	12.76	12.47
1200	19.08	19.00	19.14	18.70
1600	25.44	25.33	25.53	24.94
2000	31.79	31.66	31.91	31.17
3000	47.69	47.49	47.86	46.76
4000	63.59	63.33	63.81	62.34
5000	79.48	79.16	79.77	77.93

**Table 3:** First five natural frequencies of the force plate from finite element modal analysis.

Mode Order	Frequency (Hz)
1	206.31
2	239.05
3	244.99
4	391.19
5	501.19

**Table 4:** Resistance values of each strain gauge on the same strain beam and the output voltage of the pre-stage circuit based on structural simulation.

Force (N)	Top Strain Gauge 1 (*R*_1_) Resistance (Ω)	Top Strain Gauge 2 (*R*_4_) Resistance (Ω)	Bottom Strain Gauge 1 (*R*_2_) Resistance (Ω)	Bottom Strain Gauge 2 (*R*_3_) Resistance (Ω)	Pre-Stage Circuit Output Voltage (V)
0	350	350	350	350	2
400	350.0602	350.0599	349.9396	349.9411	2.428
800	350.1203	350.1199	349.8793	349.8820	2.856
1200	350.1805	350.1797	349.8189	349.8231	3.284
1600	350.2406	350.2396	349.7585	349.7641	3.712
2000	350.3007	350.2995	349.6981	349.7051	4.077

**Table 5:** Calibration data of the left plate.

Loading Condition	ch0 Output Voltage (V)	ch1 Output Voltage (V)	ch2 Output Voltage (V)	ch3 Output Voltage (V)
Before loading (no force)	1.4842	1.8685	1.0238	3.1105
8.587 kg placed at center	1.5026	1.8904	1.0486	3.1289
10.955 kg placed at center	1.5028	1.8917	1.0505	3.1308
18.104 kg placed at center	1.5191	1.9100	1.0727	3.1459
20.741 kg placed at center	1.5249	1.9161	1.0791	3.1501
25.491 kg placed at center	1.5350	1.9278	1.0928	3.1610
27.859 kg placed at center	1.5381	1.9281	1.0982	3.1610
37.645 kg placed at center	1.5568	1.9550	1.1222	3.1835
78.104 kg placed at center	1.6329	2.0341	1.2536	3.2646
19.841 kg placed directly above ch0	1.5662	1.8936	1.0887	3.1206
36.746 kg placed directly above ch0	1.6411	1.9088	1.1378	3.1304
19.841 kg placed directly above ch1	1.5075	1.9534	1.0247	3.1667
36.746 kg placed directly above ch1	1.5285	2.0244	1.0244	3.2115
19.841 kg placed directly above ch2	1.5374	1.8739	1.1362	3.1294
36.746 kg placed directly above ch2	1.5827	1.8784	1.2370	3.1414
19.841 kg placed directly above ch3	1.4909	1.9224	1.0538	3.1819
36.746 kg placed directly above ch3	1.4975	1.9670	1.0754	3.2419
After loading (no force)	1.4843	1.8677	1.0223	3.1142

**Table 6:** Calibration data of the right plate.

Loading Condition	ch0 Output Voltage (V)	ch1 Output Voltage (V)	ch2 Output Voltage (V)	ch3 Output Voltage (V)
Before loading (no force)	0.9081	2.6349	2.5270	2.0291
8.587 kg placed at center	0.9305	2.6522	2.5438	2.0442
10.955 kg placed at center	0.9310	2.6535	2.5420	2.0424
18.104 kg placed at center	0.9487	2.6708	2.5548	2.0564
20.741 kg placed at center	0.9553	2.6723	2.5631	2.0613
25.491 kg placed at center	0.9630	2.6858	2.5699	2.0731
27.859 kg placed at center	0.9640	2.6867	2.5705	2.0735
37.645 kg placed at center	0.9838	2.7109	2.5851	2.0931
78.104 kg placed at center	1.0676	2.7837	2.6635	2.1625
19.841 kg placed directly above ch0	0.9849	2.6522	2.5850	2.0378
36.746 kg placed directly above ch0	1.0506	2.6703	2.6336	2.0486
19.841 kg placed directly above ch1	0.9277	2.7152	2.5329	2.0717
36.746 kg placed directly above ch1	0.9417	2.7875	2.5442	2.1075
19.841 kg placed directly above ch2	0.9504	2.6399	2.5978	2.0424
36.746 kg placed directly above ch2	0.9854	2.6484	2.6565	2.0534
19.841 kg placed directly above ch3	0.9169	2.6822	2.5362	2.0828
36.746 kg placed directly above ch3	0.9222	2.7316	2.5403	2.1333
After loading (no force)	0.9057	2.6324	2.5241	2.0286

**Table 7:** Low-load static mass validation results.

Loading Condition	Reference Mass (kg)	Left Plate Measured (kg)	Right Plate Measured (kg)	Absolute Error (kg) [L/R]	MAPE (%) [L/R]
Load 1 (5 kg)	5.0	4.7	4.4	0.30/0.60	6.00/12.00
Load 2 (10 kg)	10.0	9.9	10.1	0.10/0.10	1.00/1.00
Load 3 (20 kg)	20.0	19.9	20.5	0.10/0.50	0.50/2.50
Load 4 (30 kg)	30.0	29.6	30.6	0.40/0.60	1.30/2.00
Overall Average				0.23/0.45	2.21/4.38

**Table 8:** Statistical performance evaluation of dynamic impulse measurement for bilateral force plates.

Statistical Metric	Left Force Plate	Right Force Plate
$R^{2}$ (linearity)	0.9975	0.9980
RMSE (N·s)	0.396	0.432
MAPE (%)	1.62	1.58
CV (%)	1.55	1.13

**Table 9:** Test–retest reliability of core kinetic variables and bilateral asymmetry assessment between the two testing sessions (*n* = 14).

Test Metrics	Time 1 (Mean ± SD)	Time 2 (Mean ± SD)	ICC (3, 1)	CV (%)	SEM	MDC_95_	*p*-Value (Paired *t*-Test)	Cohen’s d	Bland–Altman Mean Bias (95% LoA)
Concentric impulse (N·s)	158.49 ± 52.14	158.30 ± 51.32	0.997	1.30	2.62	7.26	0.858	−0.05	−0.18 (−7.59, 7.22)
Jump height (cm)	13.59 ± 4.24	13.43 ± 4.10	0.987	3.04	0.47	1.31	0.379	−0.24	−0.17 (−1.50, 1.17)
PF (N)	2441.56 ± 707.94	2490.70 ± 765.47	0.962	4.63	140.42	389.23	0.380	0.24	+49.14 (−347.26, 445.55)
RFD_100ms_ (N/s)	2962.18 ± 1309.63	2859.48 ± 1081.21	0.883	10.32	403.43	1118.24	0.520	−0.18	−102.70 (−1241.13, 1035.72)
ASI (%)	5.48 ± 2.73	5.62 ± 2.35	0.748	21.78	1.25	3.48	0.783	0.08	+0.14 (−3.41, 3.68)

**Table 10:** Comparison of kinetic parameters between the custom dual-plate system and the AMTI commercial force plate reference group [43].

Assessment Metrics	AMTI Commercial Force Plate Reference Group (Mean ± SD)	Custom Dual-Plate System Testing Group (Mean ± SD)	Relative Difference (%)
Jump Height (m)	0.31 ± 0.07	0.32 ± 0.04	+3.2%
Flight Time (s)	0.51 ± 0.05	0.52 ± 0.03	+2.0%
Take-off Velocity (m/s)	2.59 ± 0.25	2.48 ± 0.14	−4.2%
Concentric Impulse (N·s)	210.34 ± 41.74	200.12 ± 17.33	−4.9%
Propulsive PF (N)	2042.72 ± 343.63	1945.78 ± 194.69	−4.7%

## Data Availability

Data are contained within the article.

## Abbreviations

The following abbreviations are used in this manuscript:

GRF	Ground reaction force
CMJ	Countermovement jump
PF	Peak force
RFD	Rate of force development
ASI	Asymmetry index
AFE	Analog front-end
FEA	Finite element analysis
SJ	Squat jumps
DJ	Drop jumps
vGRF	Vertical ground reaction force
MCU	Microcontroller unit
CoP	Center of pressure
GUI	Graphical user interface
FFT	Fast Fourier transform
BW	Body weight
R^2^	Coefficient of determination
RMSE	Root mean square error
LoAs	Limits of agreement
ICC	Intraclass correlation coefficient
CV	Coefficient of variation
SEM	Standard error of measurement
MDC	Minimal detectable change
MAPE	Mean absolute percentage error
RFD_100ms_	Rate of force development at 100ms
